# Retraction: RhoB Acts as a Tumor Suppressor That Inhibits Malignancy of Clear Cell Renal Cell Carcinoma

**DOI:** 10.1371/journal.pone.0341485

**Published:** 2026-01-26

**Authors:** The PLOS One Editors

Following the publication of this article [1] concerns were raised about the results presented in Figs 1 and 5. Specifically,

- The Fig 1B RhoB and β-actin panels in [1] appear similar to lanes 1-7 of the Fig 1E RhoB and β-actin panels of [2, retracted in 3] despite differences in experimental conditions.
- The following panels appear to partially overlap:
  - Fig 5A invasion Empty vector, Fig 5A invasion Si-control Fig 5B migration Empty vector, Fig 5B migration Si-control, and Fig 5B migration Si-RhoB.
  - Fig 5A invasion RhoB and Fig 5B migration RhoB.
  - Fig 5C Empty vector 0h, Fig 5C RhoB 0h, and Fig 5C Si-RhoB 0h.

The authors did not respond to the journal’s queries pertaining to these concerns. In the absence of the original data underlying the study, these concerns cannot be resolved.

In light of the above concerns, which call into question the reliability of the published results, the *PLOS One* Editors retract this article.

SN responded but expressed neither agreement nor disagreement with the editorial decision. WC, XM, PZ, YG, YF, HP, HG, DS, LG, YZ, and XZ either did not respond directly or could not be reached.

## References

[1] Chen W, Niu S, Ma X, Zhang P, Gao Y, Fan Y, et al. RETRACTED: RhoB Acts as a Tumor Suppressor That Inhibits Malignancy of Clear Cell Renal Cell Carcinoma. PLoS One. 2016;11(7):e0157599. doi: 10.1371/journal.pone.0157599 27384222 PMC4934884

[2] Niu S, Ma X, Zhang Y, Liu Y-N, Chen X, Gong H, et al. RETRACTED: MicroRNA-19a and microRNA-19b promote the malignancy of clear cell renal cell carcinoma through targeting the tumor suppressor RhoB. PLoS One. 2018;13(2):e0192790. doi: 10.1371/journal.pone.0192790 29474434 PMC5825063

[3] The *PLOS One*Editors. Retraction: MicroRNA-19a and microRNA-19b promote the malignancy of clear cell renal cell carcinoma through targeting the tumor suppressor RhoB. PLoS One. 2018;13(2):e0192790. doi: 10.1371/journal.pone.0192790 29474434 PMC5825063

